# A Transcriptomic Biomarker Predicting Linezolid-Associated Neuropathy During Treatment of Drug-Resistant Tuberculosis

**DOI:** 10.20411/pai.v9i2.705

**Published:** 2024-06-25

**Authors:** Nika Zielinski, Dragos Baiceanu, Antonela Dragomir, Jan Heyckendorf, Elmira Ibraim, Niklas Köhler, Christoph Leschczyk, Cristina Popa, Andrea Rachow, Jens Sachsenweger, Patricia Sanchez Carballo, Dagmar Schaub, Hajo Zeeb, Begna Tulu, Andrew R. DiNardo, Christoph Lange, Maja Reimann

**Affiliations:** 1 Division of Clinical Infectious Diseases, Research Center Borstel, Borstel, Germany; 2 German Center for Infection Research (DZIF) Partner Site Hamburg-Lübeck-Borstel-Riems, Borstel, Germany; 3 Respiratory Medicine and International Health, University of Lübeck, Lübeck, Germany; 4 Marius Nasta Institute of Pneumophtiziology (MNI), Bucharest, Romania; 5 Eastern-European Study Site of DZIF in MNI, Bucharest, Romania; 6 UMF Carol Davila, Bucharest, Romania; 7 Clinic for Internal Medicine I, Leibniz Lung Clinic, University Hospital Schleswig-Holstein (UKSH) Campus Kiel, Kiel, Germany; 8 Division of Cellular Microbiology, Research Center Borstel, Borstel, Germany; 9 Division of Infectious Diseases and Tropical Medicine, Medical Centre of the University of Munich (LMU), Munich, Germany; 10 German Centre for Infection Research (DZIF), Partner Site Munich, Munich, Germany; 11 Unit Global Health, Helmholtz Zentrum München, German Research Centre for Environmental Health (HMGU), Neuherberg, Germany; 12 Department of Pneumology, Asklepios Clinic Hamburg-Harburg, Hamburg, Germany; 13 Department of Prevention and Evaluation, Leibniz Institute for Prevention Research and Epidemiology – BIPS, Bremen, Germany; 14 Faculty of Human and Health Sciences, University of Bremen, Bremen, Germany; 15 Baylor College of Medicine and Texas Children's Hospital, Global TB Program, Houston, Texas; 16 Radboud University Medical Center, Internal Medicine, Nijmegen, Netherlands

**Keywords:** adverse events, linezolid, neurotoxicity, precision medicine, *SBSN*, tuberculosis, multidrug resistance

## Abstract

**Background:**

Neuropathic adverse events occur frequently in linezolid-containing regimens, some of which remain irreversible after drug discontinuation.

**Objective:**

We aimed to identify and validate a host RNA-based biomarker that can predict linezolid-associated neuropathy before multidrug-resistant/rifampicin-resistant tuberculosis (MDR/RR-TB) treatment initiation and to identify genes and pathways that are associated with linezolid-associated neuropathy.

**Methods:**

Adult patients initiating MDR/RR-TB treatment including linezolid were prospectively enrolled in 3 independent cohorts in Germany. Clinical data and whole blood RNA for transcriptomic analysis were collected. The primary outcome was linezolid-associated optic and/or peripheral neuropathy. A random forest algorithm was used for biomarker identification. The biomarker was validated in an additional fourth cohort of patients with MDR/RR-TB from Romania.

**Results:**

A total of 52 patients from the 3 identification cohorts received linezolid treatment. Of those, 24 (46.2%) developed peripheral and/or optic neuropathies during linezolid treatment. The majority (59.3%) of the episodes were of moderate (grade 2) severity. In total, the expression of 1,479 genes differed significantly at baseline of treatment. Suprabasin (*SBSN*) was identified as a potential biomarker to predict linezolid-associated neuropathy. In the validation cohort, 10 of 42 (23.8%) patients developed grade ≥3 neuropathies. The area under the curve for the biomarker algorithm prediction of grade ≥3 neuropathies was 0.63 (poor; 95% confidence interval: 0.42 – 0.84).

**Conclusions:**

We identified and preliminarily validated a potential clinical biomarker to predict linezolid-associated neuropathies before the initiation of MDR/RR-TB therapy. Larger studies of the *SBSN* biomarker in more diverse populations are warranted.

## INTRODUCTION

According to the latest report by the World Health Organization (WHO), 10.6 million people developed tuberculosis (TB) in 2022. Second to COVID-19, TB has been the leading cause of death by an infectious disease worldwide in 2022 [1], and the emergence of drug-resistant TB is challenging control and successful treatment of this disease [2]. Based on the availability of novel drugs and treatment regimens, the treatment of drug-resistant TB has recently been revised substantially [3]. The one-armed Nix-TB Trial showed that a 3-drug regimen including bedaquiline, pretomanid, and linezolid (BPaL) administered over 6 months resulted in 90% treatment success in patients who had either failed multidrug-resistant tuberculosis (MDR-TB) treatment or had extensive drug-resistant tuberculosis (XDR-TB) [4]. However, a high rate of linezolid-associated adverse events was observed on a 1,200 mg dose administered once daily over 6 months, with 80.7% of patients experiencing polyneuropathy and 47.7% of patients experiencing bone marrow toxicity with thrombocytopenia and/or anemia.

Results from an individual patient database including 9,178 patients with multidrug-resistant/ rifampicin-resistant tuberculosis (MDR/RR-TB) indicate that linezolid is the most toxic of the second-line anti-TB medicines and that 14.1% (95% confidence interval [95%CI]: 9.9% – 19.6%) of patients receiving linezolid had to discontinue the drug due to the occurrence of adverse events [5]. Neuropathies associated with linezolid can remain irreversible if the drug is not discontinued in time, and no effective therapy has been established yet for patients who are affected [6]. With the aim to maintain the high efficacy of the BPaL regimen but to reduce the linezolid-attributed toxicity, investigators of the ZeNix-Trial evaluated standard 6-month dosages of bedaquiline and pretomanid with variable dosages and durations of linezolid over 2 and 6 months with 600 mg and 1,200 mg in 4 parallel study arms.

The best benefit-risk profile of linezolid was observed in the arm with 600 mg linezolid over 6 months. Here, 24.4% of patients still experienced neuropathies compared to 37.8% patients in the arm with 1,200 mg linezolid over 6 months [7]. In the TB-PRACTECAL study, standard dosages of bedaquiline, pretomanid, and moxifloxacin were administered with 600 mg linezolid over 16 weeks followed by 300 mg linezolid over 8 weeks with 9.3% of patients developing neuropathy [8]. As a result of these trials, the WHO has issued novel management guidelines for the treatment of drug-resistant TB in 2022 prioritizing the 6-month BPaLM treatment regimen with a linezolid dosage of 600 mg daily [3].

So far, the mechanism of action for linezolid-associated neuropathies is yet to be fully understood. Linezolid was found to inhibit autophagy flux [9] and mitochondrial protein synthesis [10], which could have an effect on the peripheral nerves by disrupting their normal function. It is important to note that a dose reduction of linezolid resulted in a decrease of attributed adverse events [7]. However, adverse events still occurred in parts of the population even at a low dose of linezolid, and it was also found that linezolid trough concentrations correlate with mitochondrial toxicity-related adverse events [11]. Adverse events, even when mild, can lead to decreased therapy adherence and consequently decreased cure rates [12]. Given this situation, it would be highly desirable to be able to predict which patients are likely to develop neuropathies when treated with linezolid. Presently, there are no biomarkers available that allow a prediction of linezolid-related adverse events in patients initiating treatment for drug-resistant TB.

Transcriptomic signatures are promising biomarkers for the prognosis of the development of TB [13, 14], diagnosis of active TB [15], and for treatment monitoring [16]. We aimed to identify and validate a biomarker model that predicts the occurrence of peripheral and/or optic neuropathy in patients receiving linezolid as part of an MDR/RR-TB treatment regimen before treatment initiation.

## METHODS

### Study Design and Participants

**Figure 1.**
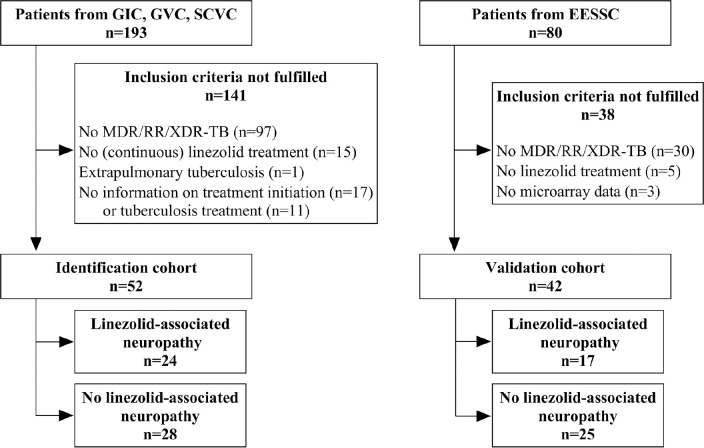
**Flow chart for study inclusion and reason for exclusion in identification and validation cohort.** GIC = German Identification Cohort; GVC = German Validation Cohort, SCVC = Second German Validation Cohort; EESSC = Eastern European Study Side Cohort; MDR/RR-TB = Multidrug-resistant/rifampicin-resistant tuberculosis; XDR-TB = Extensive drug-resistant tuberculosis

This study comprises a subgroup of patients recruited as part of an observational cohort study to evaluate various endpoints and clinical questions before, during, and after treatment of sensitive and MDR-TB with regard to transcriptome profiles for potential biomarker identification. Adult patients with culture-confirmed pulmonary MDR/RR-TB and (pre-) XDR-TB without HIV infection from 4 multicenter prospective cohort studies who received 600 mg linezolid once daily as part of a treatment regimen were included in this study (Figure 1). Three of those cohorts were enrolled in Germany: The German Identification Cohort (GIC; enrollment period: March 01, 2013 to September 30, 2016 in 5 clinical centers), the German Validation Cohort (GVC; enrollment period: March 01, 2015 to April 30, 2018 in 7 clinical centers), and the Second German Validation Cohort (SGVC; enrollment period: October 01, 2018 to July 31, 2021 at the Research Center Borstel). The fourth cohort (Eastern European Study Side Cohort [EESSC]) was enrolled between May 01, 2019 and March 31, 2023 at the Marius-Nasta Institute in Bucharest.

Sputum samples were genotypically tested for rifampicin resistance using Xpert MTB/RIF (Cepheid, Sunnyvale) and tested for second-line drug susceptibility using GenoType MTB-DRplus and MTBDRsl (Hain Life Sciences, Nehren). Phenotypic resistance testing of bacteria was additionally performed. The treatment regimen for MDR/RR-TB was individualized based on the results of genotypic and phenotypic testing of the pathogen and the current treatment guidelines [17–22]. For the entire inpatient stay as well as the follow-up period, information on the therapy regimen including antibiotics used, therapy duration (in days), therapy interruptions, and the occurrence of adverse events was captured. Clinical data of the patients, such as age, sex, time to culture positivity (TTP; MGIT liquid culture), and time to culture conversion (TCC), were also collected. At the start of treatment, all patients were tested for HIV infection.

The onset of linezolid-associated neuropathy was dated to the time when the first report of symptoms was noted in the patient's medical record or when linezolid was discontinued in response to clinical signs of neurotoxicity. Optic neuropathy was evaluated by an ophthalmologist if symptoms were stated, using fundoscopy visual accuracy testing and visual field testing. There was no standardized testing for peripheral neuropathies. Symptoms that have led to treatment discontinuation were stocking and/or glove type of sensory loss to touch and/or temperature misperception for peripheral neuropathies, and visual impairment for optic neuropathies. Severity grading was retrospectively performed by physicians using the endTB severity grading scale, version 5 [23]. Peripheral and optic neuropathies were considered together as a binary outcome variable in most analyses.

Whole blood transcriptomic RNA was collected with PAXgene tubes (Qiagen®, Venlo). In the identification cohort, PAXgene tubes were collected before the start of treatment, after 14 days of treatment, at the time of smear conversion, at the time of culture conversion, and after 6, 10, 15, and 20 months of treatment. If the treatment was longer than 20 months, another PAXgene tube was collected at the actual end of treatment. In the validation cohort, PAXgene tubes were collected before the start of treatment, after 13 days, after 8 weeks, and after 4, 6, 10, 15, and 20 months of treatment. In this study, transcriptomic RNA before the start of treatment was used only. The processing of the microarray data for the transcriptomic analyses has been described elsewhere in detail [16].

### Statistical Analysis

Patient characteristics were analyzed using absolute and relative frequencies as well as mean with standard deviation or median with interquartile range (IQR) depending on distribution. Differences between the group of patients with and without linezolid-associated neuropathies were identified through t-test, Wilcoxon rank sum test, or Fisher's exact test. The significance level was set at a *P*-value <0.05 for all analyses. The cumulative probability for linezolid-associated neuropathies during the course of treatment was calculated using a Kaplan-Meier estimator.

For the analysis of differentially expressed genes and pathways, over-representation analysis was performed by using genes with *P*-value <0.05 that were analyzed using the molecular signature of database for Hallmark, Reactome, KEGG, and Wikipathways. Ingenuity pathway analysis was used to calculate pathway z scores to clarify directionality.

Data from GIC, GVC, and SGVC were combined as the identification cohort for the development of the biomarker algorithm. The preselection of genes out of 44,000 z score standardized transcripts was done by using a LASSO regression with a binary classification for linezolid-associated neuropathy as the dependent variable. After setting up the first random forest algorithm with the randomForest package including the preselected genes from the LASSO regression, features with a mean decrease of the Gini coefficient <3 were removed. The random forest algorithm was externally validated in the EESSC cohort with the receiver operator characteristic (ROC) curve and corresponding area under the curve (AUC) including the 95%CI. Severe linezolid-associated neuropathies with a severity grade ≥3 were considered for the validation of the biomarker algorithm.

All statistical analyses were performed with R software (version 4.3.0) and GraphPad Prism (version 10.2.1).

### Ethics

The study was initially approved by the Ethics Committee of the University of Lübeck, Germany (AZ 12-233) and then approved by the local Ethic Committees of all participating centers in Germany. Study approval for the Romanian cohort was granted by the Marius Nasta Institute (3181/25.03.2015; Bucharest, Romania).

## RESULTS

**Figure 2.**
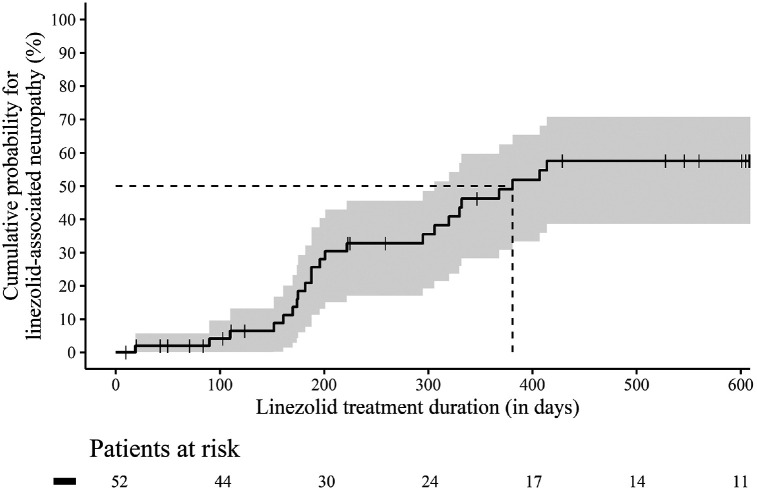
**Kaplan-Meier curve with 95% confidence interval for the cumulative probability of linezolid-associated neuropathies during the course of linezolid treatment.** The entire identification cohort (n=52) from 0 – 600 days is considered. Censored patients are shown with a cross in the curve.

In total, 52 patients were eligible for inclusion in the identification cohort (Figure 1). The median age of patients in the identification cohort was 35.5 years (IQR 26.0 – 42.0 years); 75.0% were male, and half of the patients had MDR/RR-TB (51.9%). The median TTP was 15.0 days (IQR 11.0 – 20.0 days) at baseline, the median TCC was 53.5 days (IQR 24.0 – 77.0 days), and the median linezolid therapy duration was 242.0 days (IQR 145.0 – 532.5 days). Almost three-quarters (73.1%) of the patients completed treatment with a definition of cure according to the Tuberculosis Network European Trials Group (TBNET). Six patients could not be followed up to the end of study. Patients with and without linezolid-associated neuropathies did not differ significantly with regard to socio-demographic characteristics, comorbidities, and markers for disease severity (Table 1). In the identification cohort, 24 (46.2%) patients developed linezolid-associated neuropathies. Of these, 20 patients had sensory peripheral neuropathies only, 1 patient had optic neuropathy only, and 3 patients had both sensory and optic neuropathy. Accordingly, a total of 27 events of linezolid-associated neuropathies occurred, of which 10 (37.0%) events could be assigned to severity grade 1, 16 (59.3%) events to severity grade 2, 1 (3.7%) event to severity grade 3, and 0 to severity grade 4. In all cases, therapy with linezolid was discontinued at the onset of symptoms. Linezolid-associated neuropathies occurred after a median of 198.5 days (IQR 173.0 – 330.5 days). The cumulative probability increased continuously over the course of therapy and reached over 50.0% after 381 days of linezolid treatment duration in the identification cohort (Figure 2). The validation cohort consisted of 42 patients (Figure 1), the majority of whom were male (29 patients, 69.0%), similar to the identification cohort (*P*-value = 0.644). While there were some age differences between the cohorts, with a higher median age in the validation than in the identification cohort (49.0 years [IQR 36.0 – 55.0] vs 35.5 years [IQR 26.0 – 42.0]; *P*-value <0.001), none of these characteristics differed significantly between the group of patients with and without linezolid-associated neuropathy within the validation cohort (Table 1). In the validation cohort, 17 patients (40.5%) developed linezolid-associated neuropathies, 12 patients had sensory peripheral neuropathy only, and 5 patients had a combined form of both sensory peripheral and optic neuropathy. Severity was classified on an individual patient level and not on event level in the validation cohort. Here, 3 patients were assigned to severity grade 1, 4 patients to severity grade 2, 6 patients to severity grade 3, and 4 patients to severity grade 4. Linezolid was discontinued in 6 patients.

**Table 1.** Patient Characteristics in the Identification and Validation Cohort

Identification cohort				
Characteristics	All (n=52)	Patients with linezolid-associated neuropathy (n=24)	Patients without linezolid-associated neuropathy (n=28)	*P*-value
**Age in years at baseline of treatment, median (IQR)**	35.5 (26.0 – 42.0)	41.5 (26.0 – 44.3)	31.5 (25.8 – 37.0)	0.111
**Male sex, n (%)**	39 (75.0)	18 (75.0)	21 (75.0)	1.000
**Level of resistance**				0.311
MDR/RR-TB, n (%)	27 (51.9)	14 (58.3)	13 (46.4)	
Pre-XDR-TB, n (%)	14 (26.9)	4 (16.7)	10 (35.7)	
XDR-TB, n (%)	11 (21.2)	6 (25.0)	5 (17.9)	
**TTP in days at baseline of treatment, median (IQR)** ^a^	15.0 (11.0 – 20.0)	16.0 (11.0 – 23.0)	15.0 (11.5 – 17.8)	0.554
**TCC in days at baseline of treatment, median (IQR)** ^b^	53.5 (24.0 – 77.0)	46.0 (20.0 – 69.8)	57.5 (34.0 – 88.5)	0.273
**Linezolid therapy duration in days, median (IQR)**	242.0 (145.0 – 532.5)	198.5 (173.0 – 330.5)	388.0 (103.0 – 608.0)	0.063
**Cavitary disease, n (%)**	40 (76.9)	18 (75.0)	22 (78.6)	1.000
**Bilateral disease, n (%)**	39 (75.0)	17 (70.8)	22 (78.6)	0.541
**Surgical treatment, n (%)**	5 (9.6)	2 (8.3)	3 (10.7)	1.000
**Therapy outcome (TBNET)**				0.284
Cure, n (%)	38 (73.1)	19 (79.2)	19 (67.9)	
Failure, n (%)	1 (1.9)	0 (0)	1 (3.6)	
Lost to follow-up, n (%)	6 (11.5)	1 (4.2)	5 (17.9)	
Death, n (%)	1 (1.9)	0 (0)	1 (3.6)	
Not evaluated, n (%)	6 (11.5)	4 (16.7)	2 (7.1)	
**Body-Mass-Index, mean (SD)**	21.2 (3.5)	21.4 (3.7)	21.0 (3.4)	0.605
**Diabetes mellitus, n (%)**	0 (0)	0 (0)	0 (0)	1.000
**Renal impairment, n (%)**	3 (5.8)	2 (8.3)	1 (3.6)	0.590
**Smoker, n (%)** ^c^	32 (62.7)	13 (54.2)	19 (70.4)	0.261
**Alcohol abuse, n (%)** ^c^	5 (9.8)	0 (0)	5 (18.5)	0.053

Validation cohort				
Characteristics	All (n=42)	Patients with linezolid-associated neuropathy (n=17)	Patients without linezolid-associated neuropathy (n=25)	*P*-value
**Age in years at baseline of treatment, median (IQR)**	49.0 (36.0 – 55.0)	47.0 (31.0 – 55.0)	49.0 (40.0 – 54.0)	0.617
**Male sex, n (%)**	29 (69.0)	10 (58.8)	19 (76.0)	0.314
**Diabetes, n (%)**	1 (2.4)	0 (0)	1 (4.0)	1.000
**Regular alcohol consumption, n (%)**	18 (42.9)	7 (41.2)	11 (44.0)	1.000
**Smoker, n (%)**	24 (57.1)	7 (41.2)	17 (68.0)	0.117

(a) Three missing values.

(b) Eight missing values.

(c) One missing value.

SD = Standard deviation; MDR/RR-TB = Multidrug-resistant/rifampicin-resistant tuberculosis; XDR-TB = Extensive drug-resistant tuberculosis; TTP = Time to culture positivity; IQR = Interquartile range; TCC = Time to culture conversion; TBNET = Tuberculosis Network European Trials Group.

**Figure 3.**
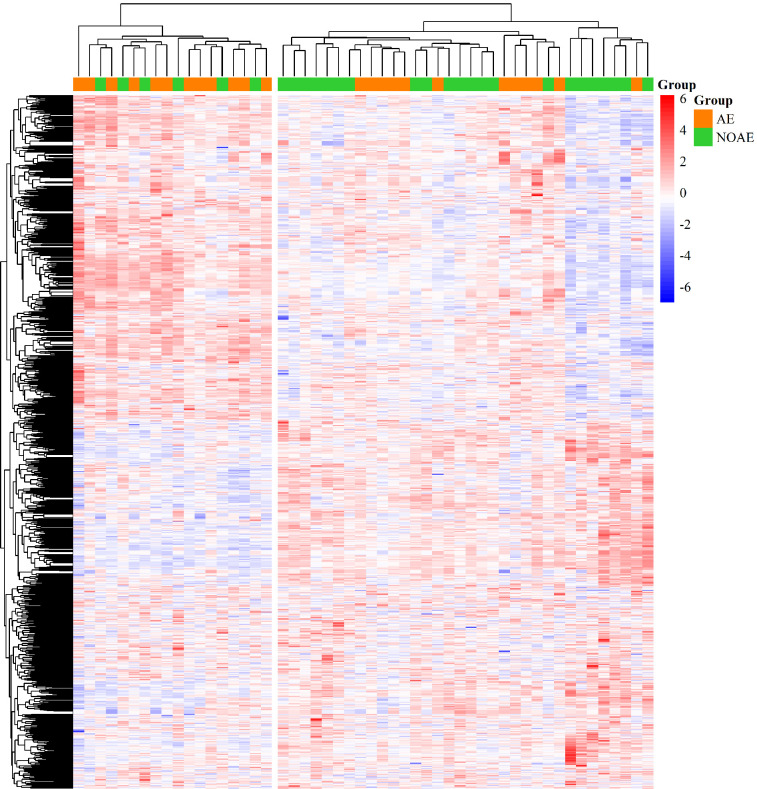
**Heat map of 1,479 statistically significant differentially expressed genes prior to treatment initiation.** The heat map shows the genes on patient-level and z score normalized. The in rows and columns are hierarchically clustered. Upregulated genes are colored red and downregulated genes are colored blue. The annotation highlights patients with (orange; AE = linezolid-associated neuropathy) and patients without linezolid-associated neuropathy (green; NOAE = no linezolid-associated neuropathy) across the top of the heat map.

In the identification cohort, 1,479 genes were statistically significant differentially expressed before treatment initiation when comparing the group of patients with and without linezolid-associated neuropathy. Two clusters were found with regard to the statistically significant differentially expressed genes that correlate with occurrence of linezolid-associated neuropathy (Figure 3).

**Figure 4.**
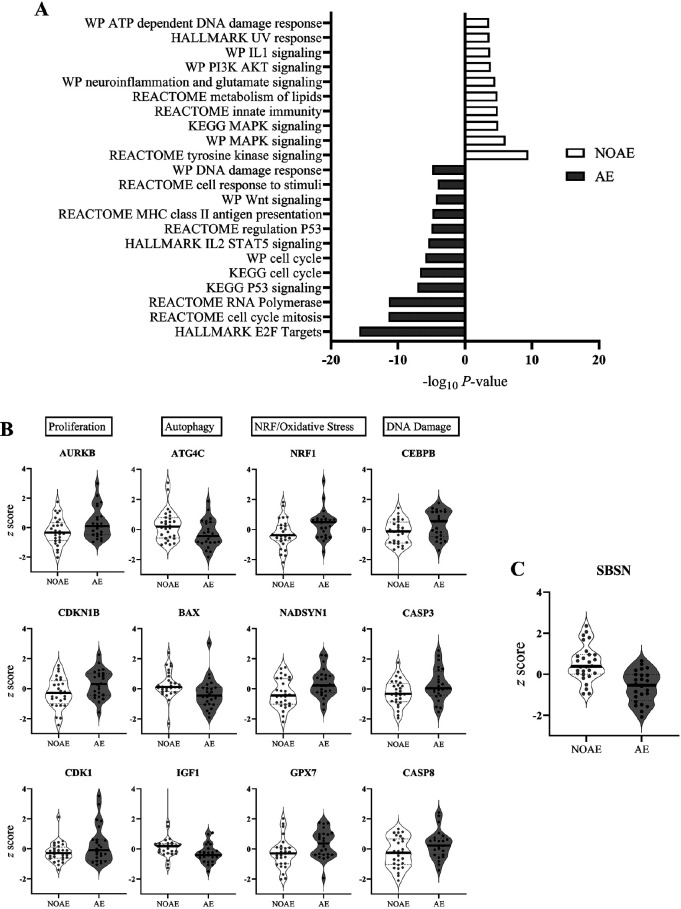
**Selected statistically significant differentially expressed pathways and genes in the group of patients with linezolid-associated neuropathy (AE) or without linezolid-associated neuropathy (NOAE).** Figure 4A. Over-representation analysis using the molecular signature of database for Hallmark, Reactome, KEGG, and Wikipathways. Figure 4B. Violin plots of z score standardized gene expression levels grouped by biological function. Figure 4C. Violin plot of the SBSN z score standardized gene expression levels.

Patients with linezolid-associated neuropathies showed a higher expression of genes associated with cellular proliferation, autophagy, Wnt signaling, p53, and immune activation. In contrast, patients without linezolid-associated neuropathy had an increase in genes associated with NRF2-mediated oxidative stress response, the ATP-dependent DNA damage response, tyrosine kinase receptor signaling, MAPK signaling, PI3K-AKT signaling, and the Il-1 pathway (Figure 4A, Supplementary Table 1). Specific genes increased in patients without linezolid-associated neuropathy included ATG4C, BAX, and IGF1, while patients on linezolid who suffered from neuropathy had an increase in AURKB, CASP3, CASP8, CDK1, CDKN1B, CEBPB, NADSYN1, NRF1, GPX7, and SBSN (Figure 4B, Figure 4C, Supplementary Table 2).

The final biomarker algorithm consists of one gene (SBSN). SBSN expression was significantly lower in patients with linezolid-associated neuropathy compared to patients without linezolid-associated neuropathy (median −0.53 vs. 0.38; *P*-value <0.001, Figure 4C). The biomarker random forest algorithm was trained in the entire identification cohort.

**Figure 5.**
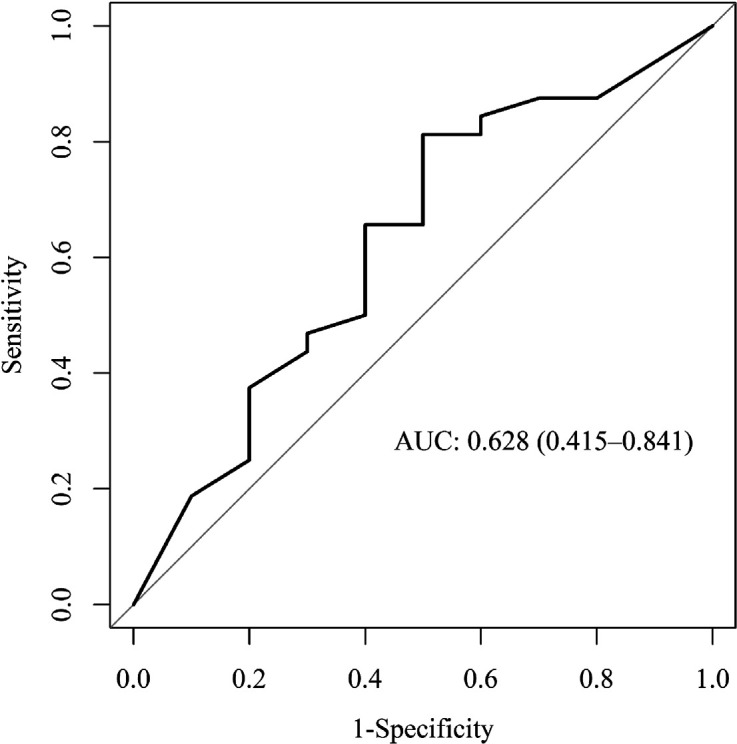
**Receiver-Operator Characteristic (ROC) for evaluation of the prediction power of the biomarker algorithm in the validation population.** Shown is the ROC curve with the area under the curve (AUC) for predicting linezolid-associated neuropathies with grade 3 or higher in the validation cohort (AUC = 0.628 [95%CI: 0.415 – 0.841]).

The discrimination for predicting severe linezolid-associated neuropathies using the biomarker algorithm in the validation cohort showed an AUC of 0.628 (95%CI 0.415 – 0.841) as displayed in Figure 5. This is considered to be poor accuracy.

Out of the 42 patients starting linezolid treatment, 32 did not develop linezolid-associated neuropathies. The biomarker algorithm correctly predicted this outcome in 21 patients corresponding to a specificity of 65.6% (95%CI 46.8% – 81.4%). Ten patients developed severe linezolid-associated neuropathies, and the biomarker algorithm predicted this correctly in 6 patients, corresponding to a sensitivity of 60.0% (95%CI 26.2% – 87.8%). Changes in sensitivity and specificity with different cut-off values as well as positive and negative predictive values for the biomarker algorithm can be found in Supplementary Table 3.

## DISCUSSION

In 4 prospective cohorts of patients undergoing treatment for drug-resistant TB with a linezolid-containing regimen, we identified and validated a transcriptomic biomarker model to predict the occurrence of neuropathies before treatment initiation. The biomarker model with SBSN predicts the occurrence of neuropathies with an accuracy of AUC = 0.628. This is, to our knowledge, the first biomarker that has the potential to predict adverse events prior to initiation of second-line anti-TB therapy, although the prediction to discriminate patients who develop neuropathies from those who do not following the initiation of a linezolid-containing regimen was still poor with this biomarker.

SBSN is a gene that is located in the q13 region of chromosome 19 and codes for the protein suprabasin. Suprabasin was first described for its function in epidermal differentiation [24]. Studies have linked suprabasin expression to human diseases such as malignant tumors [25, 26] and autoimmune diseases [27, 28]. Even though its exact function in the development of neuropathies is not established, recent findings have shown that suprabasin expression in the nervous system could play a role in neuronal function, development, and regeneration, as well as axonal growth [29, 30]. Axonal growth, as well as neuronal survival and plasticity, may be influenced through its effect on signaling pathways (AKT, WNT/β-catenin, and/or p38MAPK). These pathways are known to have an impact on several cellular processes including migration, growth, apoptosis, and immune resistance [31]. Thus, dysfunction or dysregulation of suprabasin in the nervous system could potentially contribute to the development or progression of neuropathic conditions. Suprabasin is secreted in exosomes that regulate NF-κB via NEMO [32]. Should suprabasin be activating NF-κB to induce neurotoxicity, this mechanism could possibly provide a therapeutic biomarker or target for prevention. This finding should be validated in future studies.

Comparable studies are lacking so far. In one study, expression levels of 2 genes (MKI67 and SLC22A8) were associated with neuropathies in rats receiving linezolid [33]. MKI67, a gene involved in cellular proliferation, was not increased in our study. However, our study similarly found that patients suffering from linezolid-associated neuropathy had an increase in genes related to cellular proliferation. SLC22A8 was also not increased in our study. A possible explanation could be the limited transferability from animal models to the human organism and the use of pre-treatment gene expression data in our study, whereby linezolid-based changes in gene expression after linezolid exposure were not represented. The identification of genes for the biomarker algorithm followed an exploratory, statistical approach, which offered the advantage that previously unknown genes could be identified in this context. To date, there are no studies that have emphasized SBSN in the context of drug-associated neuropathies.

The biomarker algorithm was developed in a multicenter German cohort with closely monitored clinical data and microarray transcriptomic data from standardized blood collection and laboratory preparation. The incidence of linezolid-associated neuropathies overall and the time to sensory peripheral neuropathies is comparable to other recently published cohort studies that used an individualized treatment regimen [6, 34, 35]. However, the incidence of linezolid-associated neuropathies was lower in the ZeNix-Trial (24.4%) and TB-PRACTECAL (9.3%) where the treatment duration of linezolid was shorter in the BPaL(M) regimen [7, 8]. The prediction power of the biomarker algorithm was evaluated in an additional fourth, non-German external cohort with an AUC of 0.628 (95%CI 0.415 – 0.841). If the biomarker algorithm had been applied before treatment initiation, 65.6% of linezolid-associated polyneuropathies could have been prevented.

However, in 4 out of 10 cases, the biomarker algorithm did not make the correct prediction. Based on our results, using the algorithm in clinical practice would have meant that linezolid be withheld in 34.4% of patients who did not develop a neuropathy. Close monitoring of symptoms in affected patients could therefore also be considered as an alternative recommendation. One aspect that is not considered in this biomarker algorithm but was found to be associated with the development of linezolid-associated neuropathies, are linezolid trough levels. While one study did not find statistically significant differences between trough levels of >2 mg/L and ≤2 mg/L and the incidence of linezolid-associated neuropathies [34], others found in their study that in patients with MDR-TB, the incidence of peripheral neuropathies was significantly higher in patients with a maximum trough level of >2 mg/L compared to ≤2 mg/L (46.2% vs 26.6%, *P-*value = 0.02) [6]. The combination of the biomarker algorithm with therapeutic drug monitoring could possibly fine-tune the prediction of neuropathies.

A limitation of this study is that the identified biomarker algorithm only focuses on the prediction of linezolid-associated neuropathies but does not consider other severe and potential life-threatening adverse drug reactions such as myelosuppression or lactic acidosis, which can play an important role in clinical practice. The biomarker algorithm has only been trained and preliminary validated in adult patients with pulmonary TB treated with 600 mg of linezolid once daily and not in people living with HIV. Further validation should be done in larger studies including, for example, children, elderly patients, and people living with HIV, in the BPaL(M) regimen as well as in patients from different areas of the world. In addition, an interplay of individual predisposing factors, consisting of physical and genetic characteristics, comorbidities, and lifestyle and environmental influences, can already lead to nerve damage independent of the intake of linezolid, which can occur spontaneously at the same time as taking the drug or only be unmasked by taking the drug [36]. This could limit the prediction power of the biomarker algorithm. The sample size in the identification and validation cohort is rather small. However, we would assume that the risk of overfitting is small because we used a random forest algorithm that has been shown to be suitable for dealing with OMICS data and offers the advantage that it is relatively stable against overfitting due to ensemble learning [37]. As the prediction accuracy of the SBSN biomarker remains poor at present, future studies should explore it further, including the measurement of levels from circulating blood to verify these findings.

## CONCLUSION

In conclusion, we identified and preliminarily validated a potential clinical biomarker to predict linezolid-associated neuropathies before the initiation of MDR/RR-TB therapy. Linezolid-associated neuropathies can be therapy limiting and considerably restrict the quality of life of affected patients. Early detection of linezolid-associated neuropathies can bring a decisive advantage for the prognosis of the neuropathy. Biomarker-based prediction of adverse events opens a novel avenue to improve the management of patients at risk for adverse events in the future.

## SUPPLEMENTARY DATA

Supplementary materials are available at the *Pathogens and Immunity* website. Supplementary data may be provided by the authors to benefit the reader. Supplementary data are not copyedited and are the sole responsibility of the authors. Questions or comments related to supplementary materials should be addressed to the corresponding author.
