# Supplementary material for: A Transcriptomic Biomarker Predicting Linezolid-Associated Neuropathy During Treatment of Drug-Resistant Tuberculosis
Source: Pathog Immun. 2024 Jun 25;9(2):25–42. doi: 10.20411/pai.v9i2.705 (PMC11210591; doi:10.20411/pai.v9i2.705)
Supplement: Supplementary Tables [file pai-9-025-s01.pdf]

PATHOGENS AND IMMUNITY
IN ASSOCIATION WITH CASE WESTERN RESERVE UNIVERSITY AND THE RICHARD J. FASENMYER FOUNDATION

Supplementary Table 1. Selected Human Molecular Signature Pathways Enriched in Genes Increased in Patients With and Without Linezolid-Associated Neuropathy

Gene Set Name	Pathway Database	Genes in Overlap / Genes in Gene Set	*P*-value	FDR q-value
Increased pathways in patients with linezolid-associated neuropathies				
Genes encoding cell cycle related targets of E2F transcription factors	Hallmark	23 / 200	$1.72e^{-16}$	$4.68e^{-13}$
Cell Cycle Mitotic	Reactome	30 / 560	$4.09e^{-12}$	$4.04e^{-09}$
RNA Polymerase II Transcription	Reactome	49 / 1391	$4.46e^{-12}$	$4.04e^{-09}$
Cell Cycle	Reactome	33 / 692	$7.78e^{-12}$	$5.29e^{-09}$
Genes involved in the G2/M checkpoint, as in progression through the cell division cycle	Hallmark	17 / 200	$1.96e^{-10}$	$1.07e^{-07}$
Cell Cycle Checkpoints	Reactome	18 / 291	$9.40e^{-09}$	$3.65e^{-06}$
Mitotic G1 Phase and G1/S Transition	Reactome	13 / 149	$1.96e^{-08}$	$6.68e^{-06}$
p53 Signaling Pathway	KEGG	9 / 63	$8.35e^{-08}$	$2.06e^{-05}$
Cell Cycle	KEGG	11 / 125	$2.22e^{-07}$	$4.65e^{-05}$
Transcriptional Regulation by TP53	Reactome	18 / 363	$2.64e^{-07}$	$5.13e^{-05}$
Mitotic Metaphase and Anaphase	Reactome	14 / 236	$6.87e^{-07}$	$9.87e^{-05}$
Mitotic Prometaphase	Reactome	13 / 203	$7.26e^{-07}$	$9.87e^{-05}$
Cell cycle	Wikipathways	10 / 120	$1.31e^{-06}$	$1.69e^{-04}$
Resolution of Sister Chromatid Cohesion	Reactome	10 / 126	$2.04e^{-06}$	$2.52e^{-04}$
Genes up-regulated by STAT5 in response to IL2 stimulation	Hallmark	12 / 199	$3.61e^{-06}$	$4.27e^{-04}$
Regulation of TP53 Activity through Phosphorylation	Reactome	8 / 92	$1.10e^{-05}$	$1.07e^{-03}$
MHC class II antigen presentation	Reactome	9 / 126	$1.56e^{-05}$	$1.36e^{-03}$
Network map of SARS CoV 2 signaling pathway	Wikipathways	11 / 218	$4.76e^{-05}$	$3.08e^{-03}$
Wnt signaling	Wikipathways	8 / 113	$4.91e^{-05}$	$3.11e^{-03}$
Cellular responses to stimuli	Reactome	23 / 826	$9.76e^{-05}$	$5.37e^{-03}$
Increased pathways in patients without linezolid-associated neuropathies				
Signaling by Receptor Tyrosine Kinases	Reactome	26 / 524	$3.43e^{-10}$	$9.33e^{-07}$
MAPK signaling pathway	Wikipathways	14 / 247	$8.82e^{-07}$	$3.01e^{-04}$
MAPK signaling pathway	KEGG	13 / 267	$1.12e^{-05}$	$1.69e^{-03}$
Innate Immune System	Reactome	30 / 1125	$1.24e^{-05}$	$1.78e^{-03}$
Metabolism of lipids	Reactome	23 / 746	$1.42e^{-05}$	$1.83e^{-03}$
Neuroinflammation and glutamatergic signaling	Wikipathways	9 /140	$2.96e^{-05}$	$3.35e^{-03}$
PI3K Akt signaling pathway	Wikipathways	13 / 339	$1.30e^{-04}$	$9.05e^{-03}$
Interleukin 1 IL 1 structural pathway	Wikipathways	5 / 47	$1.75e^{-04}$	$1.08e^{-02}$
Genes down-regulated in response to ultraviolet (UV) radiation	Hallmark	8 / 144	$2.25e^{-04}$	$1.30e^{-02}$
DNA damage response only ATM dependent	Wikipathways	7 / 110	$2.42e^{-04}$	$1.37e^{-02}$

Zielinski N, Baiceanu D, Dragomir A, Heyckendorf J, Ibraim E, Köhler N, Leschczyk C, Popa C, Rachow A, Sachsenweger J, Sanchez Carballo P, Schaub D, Zeeb H, Tulu B, DiNardo AR, Lange C, Reimann M. A Transcriptomic Biomarker Predicting Linezolid-Associated Neuropathy During Treatment of Drug-Resistant Tuberculosis. *Pathogens and Immunity*. 2024;9(2):25–42. doi: 10.20411/pai.v9i2.705

PATHOGENS AND IMMUNITY
In Association with Case Western Reserve University and The Richard J. Fasenmyer Foundation

Supplementary Table 2. Selected Differentially Expressed Genes. Log2 Fold Change (log2FC) Indicate Gene Expression of Patients with Linezolid-Associated Neuropathy Compared to Patients Without Linezolid-Associated Neuropathy

Gene	Gene Name	Log_2FC	*P*-value	Associated Pathways
BAX	BCL2 associated X, apoptosis regulator	-0.11	0.023	Mitochondrial Dysfunction
CASP3	caspase 3	0.20	0.050	Mitochondrial Dysfunction
CYCS	cytochrome c, somatic	0.13	0.028	Mitochondrial Dysfunction
GPX3	glutathione peroxidase 3	0.05	0.040	Mitochondrial Dysfunction
GPX7	glutathione peroxidase 7	0.17	0.027	Mitochondrial Dysfunction
MAPK9	mitogen-activated protein kinase 9	-0.09	0.043	Mitochondrial Dysfunction
MGST2	microsomal glutathione S-transferase 2	-0.15	0.047	Mitochondrial Dysfunction
NRF1	nuclear respiratory factor 1	0.09	0.018	Mitochondrial Dysfunction
PPP3CB	protein phosphatase 3 catalytic subunit beta	0.16	0.047	Mitochondrial Dysfunction
CEBPB	CCAAT enhancer binding protein beta	0.27	0.025	NAD Signaling Pathway
IGF1	insulin like growth factor 1	-0.05	0.033	NAD Signaling Pathway
NADSYN1	NAD synthetase 1	0.18	0.015	NAD Signaling Pathway
NMRK2	nicotinamide riboside kinase 2	0.04	0.002	NAD Signaling Pathway
PIK3R3	phosphoinositide-3-kinase regulatory subunit 3	0.05	0.025	NAD Signaling Pathway
CCNA2	Cyclin A2	0.08	0.036	DNA Damage/Telomere Stress Induced Senescence
CDKN1B	Cyclin Dependent Kinase Inhibitor 1B	0.22	0.026	DNA Damage/Telomere Stress Induced Senescence
BAX	BCL2 associated X, apoptosis regulator	-0.11	0.023	DNA Damage Response only ATM Dependent
MAPK9	mitogen-activated protein kinase 9	-0.09	0.043	DNA Damage Response only ATM Dependent
PIK3R3	Phosphoinositide-3-Kinase Regulatory Subunit 3	0.05	0.025	DNA Damage Response only ATM Dependent
WNT1	Wnt Family Member 1	0.03	0.014	DNA Damage Response only ATM Dependent
AURKB	aurora kinase B	0.05	0.047	
CDK1	cyclin dependent kinase 1 [	0.10	0.036	
YWHAE	tyrosine 3-monooxygenase/tryptophan 5-monooxygenase activation protein epsilon	-0.10	0.045	
AGT	Angiotensinogen	-0.02	0.044	Cachexia Signaling Pathway
CASP10	Caspase 10	-0.17	0.024	Cachexia Signaling Pathway
CASP3	Caspase 3	0.20	0.050	Cachexia Signaling Pathway
CASP8	Caspase 8	0.24	0.044	Cachexia Signaling Pathway
IGF1	Insulin Like Growth Factor 1	-0.05	0.033	Cachexia Signaling Pathway
IL2	Interleukin 2	0.03	0.050	Cachexia Signaling Pathway
MAP2K4	Mitogen-Activated Protein Kinase Kinase 4	-0.29	0.011	Cachexia Signaling Pathway
MAPK9	Mitogen-Activated Protein Kinase 9	-0.09	0.043	Cachexia Signaling Pathway
TNFSF15	TNF Superfamily Member 15	-0.03	0.019	Cachexia Signaling Pathway
CBR1	Carbonyl Reductase 1	-0.23	0.036	NRF2-mediated Oxidative Stress Response
HSPB8	heat shock protein family B (small) member 8	-0.03	0.047	NRF2-mediated Oxidative Stress Response

Zielinski N, Baiceanu D, Dragomir A, Heyckendorf J, Ibraim E, Köhler N, Leschczyk C, Popa C, Rachow A, Sachsenweger J, Sanchez Carballo P, Schaub D, Zeeb H, Tulu B, DiNardo AR, Lange C, Reimann M. A Transcriptomic Biomarker Predicting Linezolid-Associated Neuropathy During Treatment of Drug-Resistant Tuberculosis. *Pathogens and Immunity*. 2024;9(2):25–42. doi: 10.20411/pai.v9i2.705

MAP2K4	Mitogen-Activated Protein Kinase Kinase 4	-0.29	0.011	NRF2-mediated Oxidative Stress Response
MAPK9	Mitogen-Activated Protein Kinase 9	-0.09	0.043	NRF2-mediated Oxidative Stress Response
MGST2	microsomal glutathione S-transferase 2	-0.15	0.047	NRF2-mediated Oxidative Stress Response
PIK3C2G	Phosphatidylinositol-4-Phosphate 3-Kinase Catalytic Subunit Type 2 Gamma	-0.03	0.003	NRF2-mediated Oxidative Stress Response
PIK3R3	Phosphoinositide-3-Kinase Regulatory Subunit 3	0.05	0.025	NRF2-mediated Oxidative Stress Response

Supplementary Table 3. Sensitivity and Specificity and Corresponding 95% Confidence Interval (95%CI) with Changing Cut-off Values for the Classification Based on the Prediction Value for Linezolid-Associated Neuropathies Using the Biomarker Algorithm.

Cut-off Value	Sensitivity (95%CI)	Specificity (95%CI)	Positive Predictive Value (95%CI)	Negative Predictive Value (95%CI)
0.1	80.0% (44.4% – 97.5%)	37.5% (21.1% – 56.3%)	28.6% (13.2% – 48.7%)	85.7% (57.2% – 98.2%)
0.2	60.0% (26.2% – 87.8%)	50.0% (31.9% – 68.1%)	27.3% (10.7% – 50.2%)	80.0% (56.3% – 94.2%)
0.3	60.0% (26.2% – 87.8%)	53.1% (34.7% – 70.9%)	28.8% (11.3% – 52.2%)	80.9% (58.1% –94.6%)
0.4	60.0% (26.2% – 87.8%)	65.6% (46.8% – 81.4%)	35.3% (14.2% – 61.7%)	84.0% (63.9% – 95.5%)
0.5	60.0% (26.2% – 87.8%)	65.6% (46.8% – 81.4%)	35.3% (14.2% – 61.7%)	84.0% (63.9% – 95.5%)
0.6	60.0% (26.2% – 87.8%)	65.6% (46.8% – 81.4%)	35.3% (14.2% – 61.7%)	84.0% (63.9% – 95.5%)
0.7	50.0% (18.7% – 81.3%)	71.9% (53.3% – 86.3%)	35.7% (12.8% – 64.9%)	82.1% (63.1% – 93.9%)
0.8	50.0% (18.7% – 81.3%)	81.3% (63.6% – 92.8%)	45.5% (16.7% – 76.6%)	83.9% (66.3% – 94.5%)
0.9	40.0% (12.2% – 73.8%)	84.4% (67.2% – 94.7%)	44.4% (13.7% – 78.8%)	81.8% (64.5% – 93.0%)
